# Effect of pyrolysis temperature on sulfur content, extractable fraction and release of sulfate in corn straw biochar

**DOI:** 10.1039/c8ra06382f

**Published:** 2018-10-18

**Authors:** Baowei Zhao, Huan Xu, Tao Zhang, Xujun Nan, Fengfeng Ma

**Affiliations:** School of Environmental and Municipal Engineering, Lanzhou Jiaotong University Address: No. 88, West Anning Road Lanzhou 730070 Gansu P. R. China baowei.zhao@yahoo.com zhbw2001@sina.com +86-931-4956017 +86-931-4938017

## Abstract

The contents and release of the nutrient elements N, P and K in biochars have been investigated. Sulfur is an indispensable element for plants, but its content and release in biochar are still unclear. The effect of pyrolysis temperature (300, 500 and 700 °C) on the sulfur content, extractable fraction and release of sulfate in corn straw biochars (CS300, CS500 and CS700) was investigated. The biochars were characterized using element analysis, BET, FTIR, and XRD. It was shown that the contents of sulfur in biochars decreased significantly with increasing pyrolysis temperature. The extraction results indicated that the percentages of water extractable-sulfate (W–SO_4_^2−^) and organosulfur in biochars decreased while those of HCl- and NaH_2_PO_4_-extractable sulfate (HCl–SO_4_^2−^, NaH_2_PO_4_–SO_4_^2−^) increased with pyrolysis temperature. Batch release experiments were conducted to test the effect of contact time and addition of Hoagland nutrient solution (HNS) on the release of sulfate from biochars. The release kinetics fitted well with a pseudo-second-order model. Approximately 10.7 mg g^−1^ of sulfate was released from CS300 during the initial 2 h, whereas 6.32 and 3.93 mg g^−1^ were released from CS500 and CS700, respectively. Increasing the amounts of HNS led to negative effects on sulfate release. The results indicate that low-temperatures might be optimal for producing biochar from corn straw to improve the sulfur fertilization.

## Abstract 2

Low pyrolysis temperature is optimal for biochar to release sulfate and the release kinetics fitted well with a pseudo-second-order model.

## Graphical abstract

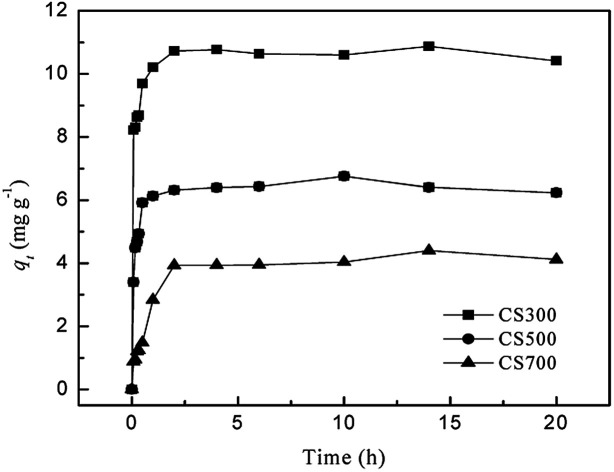

## Introduction

Sulfur is an essential and growth-limiting plant nutrient. It is required for protein synthesis, occurs in the form of sulfolipids in photosynthetic membranes and indirectly affects the efficiency of the plant in the use of other plant nutrients.^1–3^ Sulfur plays a key role in processes regulating plant growth, detoxification, defense and resistance. In fact, more recently, sulfur deficiency in plants has been reported in several parts of the world.^4^ Industrial emission laws have gradually decreased the amount of sulfur released into the atmosphere in past decades. This, combined with a growing trend toward non-sulfur based fertilizers, is causing sulfur deficiency in many crops planted in lighter and sandier soils.^5^ The increased sulfur removal from soils under intensive cropping systems and increased crop yields have contributed to sulfur deficiency in plants. As a result, it is becoming increasingly important that more is known about the additives that influence sulfur utility in soils.

Biochar is a by-product of biomass pyrolysis in which the plant-derived materials are heated in the absence of oxygen. It has been suggested as a means to combat climate change and to achieve agricultural and environmental benefits.^6–8^ If biochar can be used as a soil amendment to improve soil quality and to increase crop production, an obvious positive attribute of biochar is its nutrient value, supplied either directly by providing nutrients to plants or indirectly by improving soil properties, with a consequent improvement in the efficiency of fertilizer use.^9^ Direct nutrient values and the availability of biochar depend on the biomass feedstock and pyrolysis conditions.^9,10^ Numerous studies have reported that the addition of biochar to soils could indirectly increase the bioavailability of soil nutrients and improve crop production, such as nitrogen (NH_4_^+^ and NO_3_^−^), phosphorus (PO_4_^3−^) and potassium (K^+^).^11–14^ A few studies have even investigated the potential direct nutrition values of nitrogen and phosphorous in biochars,^15–25^ where the pyrolysis temperature,^15,18–20,23,25^ source and quality of biomasses^20^ and nutrient composition in biochars^15,16,22,24,25^ were concerned. However, there have been few reports concerning the indirect and direct values of sulfur in biochars,^26–28^ to our knowledge.

Being similar to other nutrient elements such as nitrogen and phosphorous, it is predicted that biochar itself may be a potential source of sulfur. Direct nutrient values and availability of sulfur in biochars could also depend on pyrolysis conditions according to the present literature. This may be related to changes in the composition of biochar and speciation of sulfur in biochar as the temperature of biochar production changes. Cheah *et al.* reported that corn stover biochars produced under pyrolysis conditions at 500–600 °C contain sulfate, organosulfur, and sulfide.^28^ Knudsen *et al.* found that 35–50% of the total sulfur was released to the gas phase during the herbaceous biomass volatilization at 400 °C. Sulfur forms clearly varied as the temperature of the thermal conversion from 500 to 700 °C.^26^ Blum *et al.* found that speciation rather than the total sulfur of plant litter and corn stover biochar is likely the major factor that influences sulfur mineralization in soil.^27^ These limited results generate interest in finding the quantity of sulfur speciation in biochars produced under different pyrolysis conditions. Moreover, the release of sulfate from biochar produced under different pyrolysis conditions is currently unclear.

In this paper, the biochars were prepared from corn straw at 300, 500 and 700 °C and characterized using BET, element analysis, FTIR and XRD. The sulfur content, extractable fraction and release of sulfate in biochars were investigated. The overall objectives of the present study are to investigate (i) the influence of pyrolysis temperature on the sulfur content and extractable sulfate fraction in biochars; (ii) the dynamics of SO_4_^2−^ release and the effects of other nutrient elements on SO_4_^2−^ release from biochars. This investigation can provide detailed information to enable a better understanding of the properties of biochar to be chosen for soil fertilization with sulfur.

## Materials and methods

### Chemicals and materials

Analytical grade sodium sulfate was purchased from Shanghai Chemical Co., China. Deionized water was used in all of the experiments.

The corn straw (CS) was collected from the rural land in Tianshui City, China. It was washed with tap water, and air-dried for one week, then crushed into particles using a grinder (FW100, Tianjin Hauxing Instrument Company, China). The element composition of CS is listed in Table 1. The powdered corn straw was tightly placed into a crucible. The filled crucible was covered with a lid. Then, the samples were placed in a muffle furnace (SX2, Shanghai Yuejin Medical Instrument Factory, China) to pyrolyze the biomass under an oxygen limited atmosphere. The pyrolysis temperatures were designed as 300, 500 and 700 °C respectively and the pyrolysis duration was designed as 6 h. After cooling, the obtained biochar was passed through a 0.18 mm sieve and stored under ambient conditions for the subsequent experiments. The biochars were labeled as CS300, CS500 and CS700.

**Table 1:** Physical and chemical properties of CS300, CS500 and CS700 on dry basis

Property	CS	CS300	CS500	CS700
**Composition (%)**				
C	42.61	58.83	61.75	66.39
H	6.37	3.72	1.92	0.86
O	41.77	21.21	13.52	9.88
N	1.23	2.21	1.68	1.30
S	0.97	0.58	0.46	0.32
Ash	7.05	13.45	20.68	21.25

**Atomic ratio**				
O/C	0.75	0.36	0.22	0.15
H/C	1.79	0.06	0.03	0.01
(O + N)/C	0.78	0.40	0.25	0.17
pH	—	9.25	9.88	10.45
*S*_BET_ (m^2^ g^−1^)	—	1.70	7.70	209

### Characterization

The total C, N, H, O and S in biochar were determined with an elemental analyzer (Vario EL, Elementar, Germany). The pH of each biochar was measured using a pH meter with a 1 : 2.5 (w/w) suspension of the biochar in deionized water. Brunauer–Emmett–Teller (BET) surface areas (*S*_BET_) were obtained from N_2_ adsorption at 77 K using a Quantachrome Autosorb-1 (Quantachrome, USA). Organic functional groups present on the surface of biochar were determined by Fourier-transform infrared spectroscopy (FTIR) (NEXUS 670, Thermo Nicolet, USA). X-ray diffraction (XRD) patterns were obtained using a PANalytical instrument (Netherlands) with Cu Kα radiation at 40 mA and 40 kV.

### Sulfate fraction extraction

We used a wet chemical method similar to that described in ASTM D2492 for the analysis of forms of sulfur.^29^ The inorganic sulfate contents could be detected by these analytical procedures, thus the organic sulfur fraction was calculated by the difference between total sulfur and inorganic sulfur.^28^ Three different extraction procedures were utilized in the detection of the inorganic sulfur: 2.50 g biochar and 15 mL of deionized water were placed into a centrifuge tube. The tube was shaken at 145 rpm and 25 ± 1 °C on a reciprocating shaker (CHA-S Shaker, Jintan Danyang Instrumental Company, China) for 1 h. The mixture was centrifuged at 4000 rpm for 15 min. The supernatant was removed and sulfate in it was analyzed as water extraction sulfur (W–SO_4_^2−^). Then 15 mL of 0.025 mol L^−1^ NaH_2_PO_4_ was added into the tube. After the same shaking and centrifugation as above, the supernatant was removed and sulfate in it was analyzed as NaH_2_PO_4_ extraction sulfur (NaH_2_PO_4_–SO_4_^2−^). At last, 15 mL of 0.5 mol L^−1^ HCl was added into the tube. After the same shaking and centrifugation as above, the supernatant was removed and sulfate in it was analyzed as HCl extraction sulfur (HCl–SO_4_^2−^). The extraction solution was filtered with a 0.45 μm membrane and measured for SO_4_^2−^ amount by indirect atomic absorption spectrometry (AAS).^30^

### Sulfate release kinetics

A biochar sample (0.050 g) was placed in a 100 mL Erlenmeyer flask, and 50 mL of deionized water was added. After being shaken at 145 rpm and 25 ± 1 °C on a reciprocating shaker (CHA-S Shaker, Jintan Danyang Instrumental Company, China), the mixture was removed at appropriate time intervals and then filtered with a 0.45 μm membrane. The concentration of SO_4_^2−^ in each of the supernatants was determined by indirect AAS. Each sample was prepared in triplicate. The amount of SO_4_^2−^ released from the biochar *q* (mg g^−1^) was calculated from the difference between the initial and equilibrium concentrations of SO_4_^2−^ (mg L^−1^) in solutions, together with the volume of solution (L) and the mass of biochar (g).

### Effect of Hoagland nutrient solution on sulfate release

To study the effect of other nutrient elements on sulfate release, biochar was explored in a similar way at different rates (10%, 20%, 30%, 40% and 50%) of modified Hoagland nutrient solutions (HNS). The 100% modified HNS comprised 4 mmol L^−1^ Ca(NO_3_)_2_, 6 mmol L^−1^ KNO_3_, 4 mmol L^−1^ KH_2_PO_4_, and 1 mmol L^−1^ NH_4_Cl. The results are expressed as the average of three replicates.

## Results and discussion

### Characterization of biochar

The physicochemical properties of the biochars are shown in Table 1. All properties were strongly influenced by the temperature of pyrolysis. The specific surface areas (*S*_BET_) of biochars are significantly different and increased in the order of magnitude with pyrolysis temperature, with the values 1.70, 7.70 and 209 m^2^ g^−1^ for CS300, CS500 and CS700. The ash content of biochar increased with increasing pyrolysis temperature from 13.45% for CS300 to 20.68 and 21.25% for CS500 and CS700 due to the abundance of mineral elements in corn stalk.^31,32^ The pH values of biochars exhibited a positive correlation with increasing temperature and ash content. The carbon content remaining in the biochars during pyrolysis increased with temperature. However, the hydrogen, oxygen, nitrogen and sulfur contents decreased. These trends are common with increasing temperature of pyrolysis.^32–34^ As the pyrolysis temperature increased, the atomic ratios of H/C, O/C, and (O + N)/C decreased, which indicates that the aromaticity of biochars enhanced while polarity decreased.^35^ The conversion of aliphatic C to aromatic C during pyrolysis is accompanied by a reduction in C mineralization rates. This reduction in mineralization of organic C also suggests a reduction in the availability of nutrients in biochar that are bound in the organic structure, such as N, P and S.^9^

As seen in Table 1, the sulfur contents decreased from 0.58% for CS300 to 0.46% for CS500 and 0.32% for CS700 with pyrolysis temperature increasing. Cantrell *et al.* and Al-Wabel *et al.* also reported decreases in sulfur content with temperature, probably due to loss of sulfur by sulfur-containing volatile organic compounds at 350 °C.^36,37^ However, Liu *et al.* and Devi and Saroha observed that sulfur content in biochars remained almost stable throughout the temperature range 300–700 °C.^38,39^ Contrarily, sulfur content increased with increasing pyrolysis temperature was observed for cotton crop residues biochars prepared at 300, 400, 500 and 700 °C, which mainly due to low sulfur-containing volatile compounds and/or organic compounds being resistant to sulfur-bond breakages by temperature, and formation of mineral sulfates.^32^

**Fig. 1:**
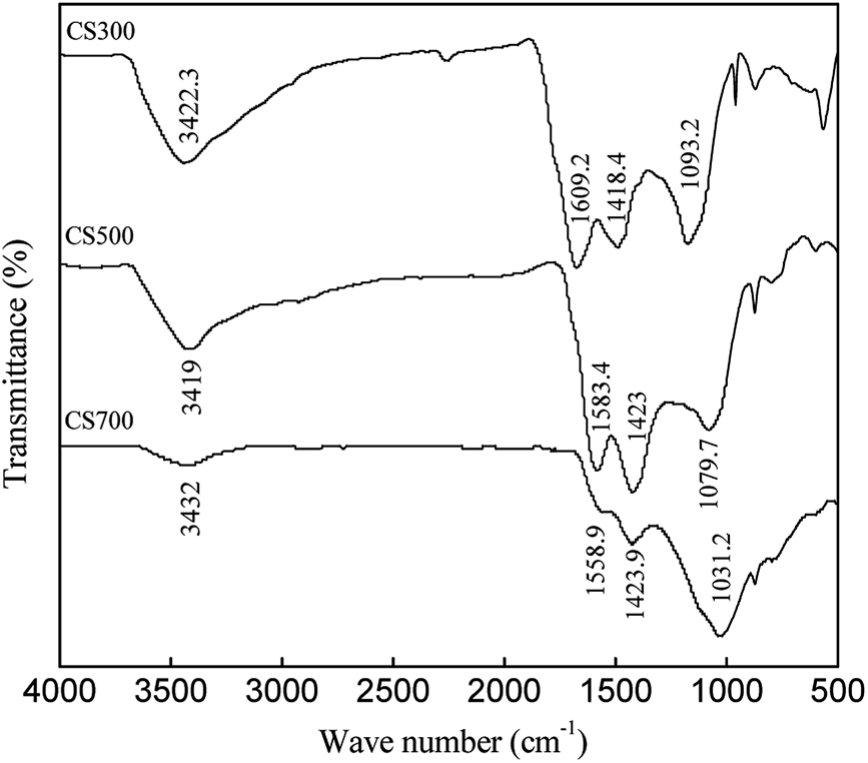
FTIR spectra of CS300, CS500 and CS700.

The FTIR spectra of CS300, CS500 and CS700 are presented in Fig. 1. It is obvious that the peak characteristics of main functional groups in biochars changed with pyrolysis temperature. The peaks at about 3400 cm^−1^ associated with O–H stretching vibration decreased with pyrolysis temperature. There was stronger C=O stretching vibration at 1600 cm^−1^, indicating the formation of ketones, quinones, and carboxylic C.^32^ However, it declined in CS500 and disappeared in CS700. The peaks at approximately 1420 cm^−1^ could be attributed to the C=C such as olefince and aromatic ring. At the same time the wave numbers of the peaks at 1030–1110 cm^−1^ associated with dominant C–O stretching shifted significantly, suggesting degradation of biomass.^32,36^ The above results indicates that conversion of aliphatic C to aromatic C in biochars occurred when the pyrolysis temperature increased.

**Fig. 2:**
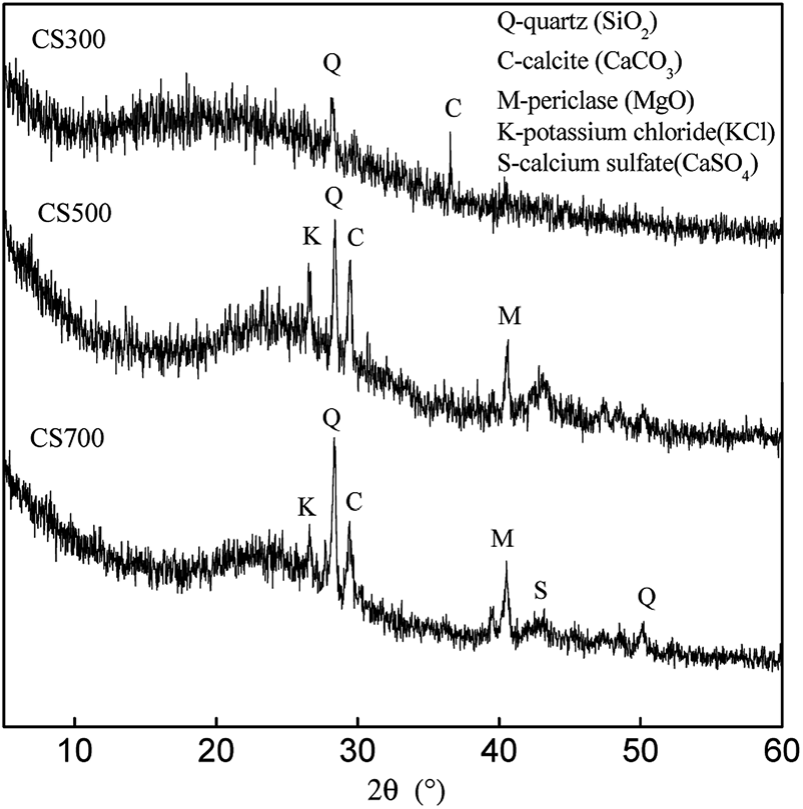
XRD spectra of CS300, CS500 and CS700.

The XRD spectra of the biochars are shown in Fig. 2. For CS300, only the presence of SiO_2_ was confirmed by peaks at 2*θ* = 28.244° and calcite was confirmed by peaks at 2*θ* = 36.59°. However, when the pyrolysis temperature was increased, there were more sharp small diffraction peaks for CS500 and CS700, which showed a very well crystal structure and a high content of mineral components. The intense sharp peak at 2*θ* = 29.485° indicated that calcite was well crystallized where CaCO_3_ was formed. A weak peak at 2*θ* = 43.18° showed the formation of calcium sulfate (CaSO_4_). This indicates that the increase of pyrolysis temperature is accompanied by the increase of the insoluble sulfate content and salts content in the biochar. The similar results were also obtained for phosphorous in biochars. Bekiaris *et al.* found that an increase in the pyrolysis temperature led to the formation of a large variety of P species in biochars. Hydroxyapatite and tricalcium phosphate were the most dominant P species in the mid to high temperature range (600–900 °C).^22^ Bruun *et al.* found that the primary species of P in bichars derived from solid fraction of manure were simple calcium phosphates.^25^

### Extraction forms in biochars

**Fig. 3:**
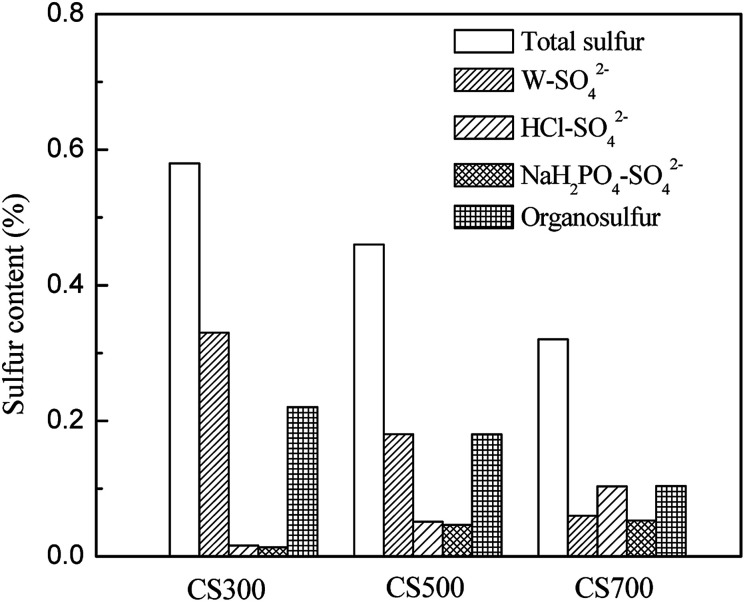
Contents of total sulfur, extractable sulfate and organosulfur in CS300, CS500 and CS700.

Fig. 3 shows the distribution of total sulfur content, extractable sulfate and organic sulfur in CS300, CS500 and CS700. With pyrolysis temperature increasing and total sulfur in biochars decreasing, the content of W–SO_4_^2−^ decreased while those of HCl–SO_4_^2−^ and NaH_2_PO_4_–SO_4_^2−^ increased, which can further illustrate the results from XRD. The contents of water-soluble SO_4_^2−^ (W–SO_4_^2−^) decreased from 0.33% in CS300 to 0.18% in CS500 and then to 0.06% in CS700. In addition, in CS300 and CS500, the sulfur contents of W–SO_4_^2−^ were much higher than those of HCl–SO_4_^2−^. This could be attributed to the effects of pyrolysis temperature on sulphur form in biochars. Knudsen *et al.* studied sulphur transformation during pyrolysis of typical Danish wheat straw. Before pyrolysis, sulphur was found to be associated as inorganic sulphate (40 to 50 per cent of total sulfur) and partly as proteins (50 to 60 per cent). At lower temperatures (400 °C), 35–50 per cent of the total sulphur was released to the gas phase as a result of decomposition of organic sulphur while inorganic sulphate decreased slightly. At higher temperatures (500 °C to 700 °C), the residual sulphur contents of biochar did not change significantly. However, the forms of sulphur changed, with a disappearance of inorganic sulphate due to the conversion to insoluble sulphide or from fixed to reactive biochar surfaces by either addition of sulphur to unsaturated sites or by substitution of oxygen in surface oxides.^26^ The results reported by Cheah *et al.* indicated that there is more sulfate in the corn stover 500 °C biochar than the corn stover 850 °C biochar even when accounting for sample heterogeneity, *i.e.* 63 and 17% sulfate species for the corn stover biochar produced at 500 and 850 °C, respectively. The elements in biomass that are likely bound to sulfur in the sulfate form are Ca, K, Mg, and Na.^28^ According to thermodynamic calculations published in the literature, silicate may compete with sulfate for these cations.^40^ Therefore, even though there are high concentrations of metals, such as potassium and calcium in corn stover, they may not be able to capture much of the sulfur. This would result in more sulfur being released to the syngas, a phenomenon often reported for herbaceous feedstocks.^26,40^ Because the stability of silicate is higher than that of sulfate at higher temperatures, silicate is able to sequester even more of the inorganic cations, resulting in a lower W–SO_4_^2−^ concentration in CS700 and CS500 than in the CS300.

The different forms of sulfur in biochar led to different percentages released. Low-temperature biochar has more sulfate that is immediately and more readily available to the plant. Schneider and Haderlein found that in two slightly acidic hydrochars derived from sewage sludge, most P was associated with Fe and was extractable by 0.1 mol L^−1^ NaOH while in the remaining alkaline pyrochars most P was associated with Ca and was extractable by 1 mol L^−1^ HCl.^24^ Mukherjee and Zimmerman found that the release of N and P from biochars into water was correlated with both the volatile-matter content and acid functional-group density of biochars.^16^ Bruun *et al.* also found that the low availability of P in the biochar produced at high temperatures can likely be explained by the formation of less-soluble P species in the biochar.^25^

The organic sulfur decreased from 0.22% in CS300 to 0.16% in CS500 and then to 0.1% in CS700 (Fig. 3). Much of the organosulfur was believed to be found in the proteins. The decomposition temperatures of the sulfur-bearing amino acids such as cysteine and methionine are 178 and 283 °C, respectively.^41^ It is further suggested that the initial sulfur release is caused predominantly by decomposition of organosulfur. The contents of different forms of sulfur may be ruled by a temperature-dependent pattern, which suggests that the pyrolysis temperature is one of the determining factors of sulfur speciation in the biochar.

### Effect of time on sulfate release

**Fig. 4:**
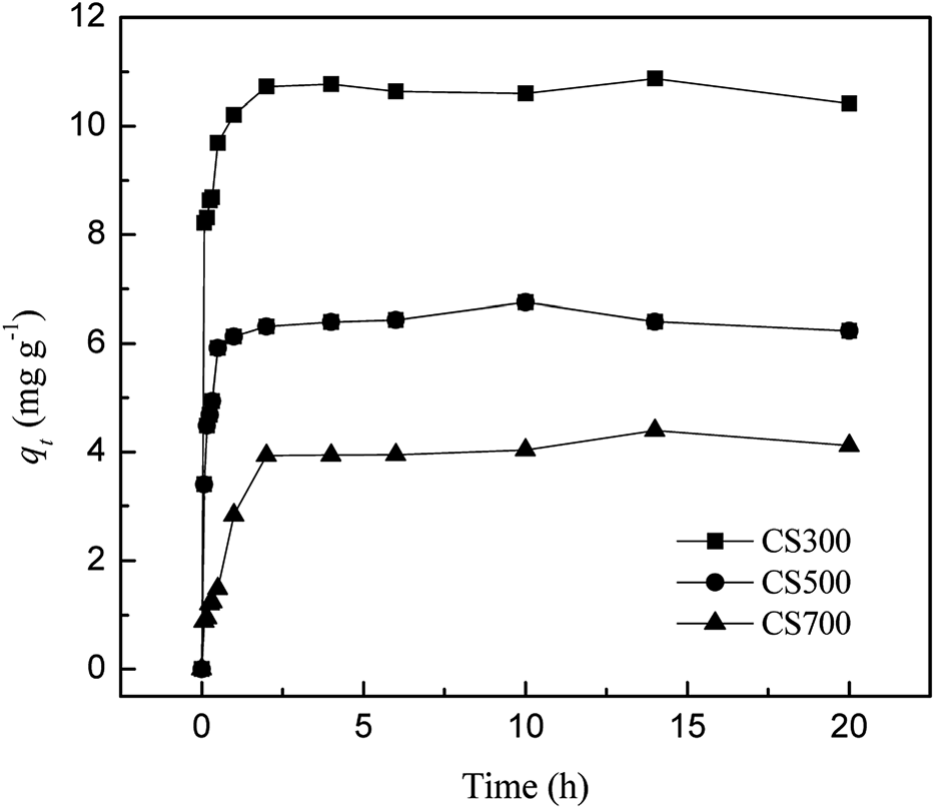
Plots of released amounts of SO_4_^2−^ in CS300, CS500 and CS700 *versus* time.

The results of SO_4_^2−^ release kinetics experiments on biochars are shown in Fig. 4. The SO_4_^2−^ release from biochars mainly occurred within 2 h, followed by a slow increase (less than 5%) from 2 to 20 h, showing that these biochars contain rapidly released SO_4_^2−^. Approximately 10.7 mg g^−1^ of sulfate was released from CS300 in the initial 2 h, whereas 6.32 and 3.93 mg g^−1^ were released from CS500 and CS700. After 2 h, the release of SO_4_^2−^ levelled off. The similar pattern was also observed by Qian *et al.*, for P release from biochars made from rice husks. When used in field situations, the SO_4_^2−^ forms supplied by the biochars prepared at low temperature would be readily available for plant uptake.^17^ Thus, these biochars could be useful to augment crop fertilizer needs. Mukherjee and Zimmerman found that, in successive batch extractions of biochar, cumulative losses were approximately 0.1–2, 0.5–8 and 5–100% of the total C, N and P initially present, respectively, with greater releases from biochars made from grass at lower temperature.^16^

Using pseudo-first-order (1), pseudo-second-order (2), and power-law (3) models, the release kinetics of SO_4_^2−^ from biochars are examined and can be expressed by the following equations:

1 ln(*q*_e_ − *q*_*t*_) = ln *q*_e_ − *k*_1_*t*

2 *t*/*q*_*t*_ = 1/*k*_2_*q*_e_^2^ + *t*/*q*_e_

3 *q*_*t*_ = *at*^*b*^

where *q*_e_ and *q*_*t*_ (mg g^−1^) are the release capacities at equilibrium and at time *t* (h), respectively; *k*_1_ (h^−1^), *k*_2_ (g mg^−1^ h^−1^), *a* ((mg g^−1^ h^−1^)^*b*^) and *b* (mg^−1^ g^−1^) are constants that relate to the concentration of SO_4_^2−^ released.

The kinetic data were fitted using three kinetic models to understand the processes governing SO_4_^2−^ release from biochars (Table 2). The *R*^2^ values of the pseudo-second-order model for release of SO_4_^2−^ from biochars ranged from 0.987 to 0.997, which are higher than those of the power model and the pseudo-first-order model. Moreover, the calculated release capacity values of SO_4_^2−^ (*q*_e,cal_) from the pseudo-second-order model are closer to the experimental results (*q*_e,exp_). Thus, the pseudo-second-order model provides the best fit with the experimental data. This suggests that the rate-limiting step of SO_4_^2−^ release might be the chemical interaction between sulfur and the biochar surface. The correlation coefficients (*R*^2^) for the power function were all above 0.8 and can be a good choice for describing the release kinetics of SO_4_^2−^ from biochars. The two parameters of the power function can be viewed as indicators of the initial concentration of SO_4_^2−^ release (*a*) and the rate at which the release of nutrients declines with time (*b*).^42^ Generally, for SO_4_^2−^, the parameters *a* and *b* decreased and increased with temperature, respectively, suggesting that lower amounts of SO_4_^2−^ were released from biochars produced at high temperatures. Biochars containing mineral matter (ash) produced at low temperatures have a much greater concentration of sub-grain boundaries and defects on the surface than the same biochars produced at high temperatures. Mineral matter in low-temperature biochars is more likely to dissolve since these defects are centers for reactions with liquids and gases. These changes should have effects on the total nutrient content as well as their availability.^9^

**Table 2:** Kinetic model parameters for SO_4_^2−^ releasing from biochars

Parameter		CS300	CS500	CS700
*q*_e,exp_ (mg L^−1^)		10.82	6.08	4.03
Pseudo-first-order	*k*_1_ (h^−1^)	0.18	0.22	1.10
	*q*_e,cal_ (mg L^−1^)	3.52	1.98	3.81
	*R* ^2^	0.735	0.957	0.943
Pseudo-second-order	*k*_2_ (g mg^−1^ h^−1^)	0.85	2.30	5.53
	*q*_e,cal_ (mg L^−1^)	10.86	6.59	4.25
	*R* ^2^	0.988	0.997	0.987
Power	*a* (mg g^−1^ h^−1^)^*b*^	2.75	2.01	1.41
	*b* (mg^−1^ g^−1^)	0.24	0.36	0.68
	*R* ^2^	0.814	0.843	0.852

### Effect of Hoagland nutrient solution on sulfate release

**Fig. 5:**
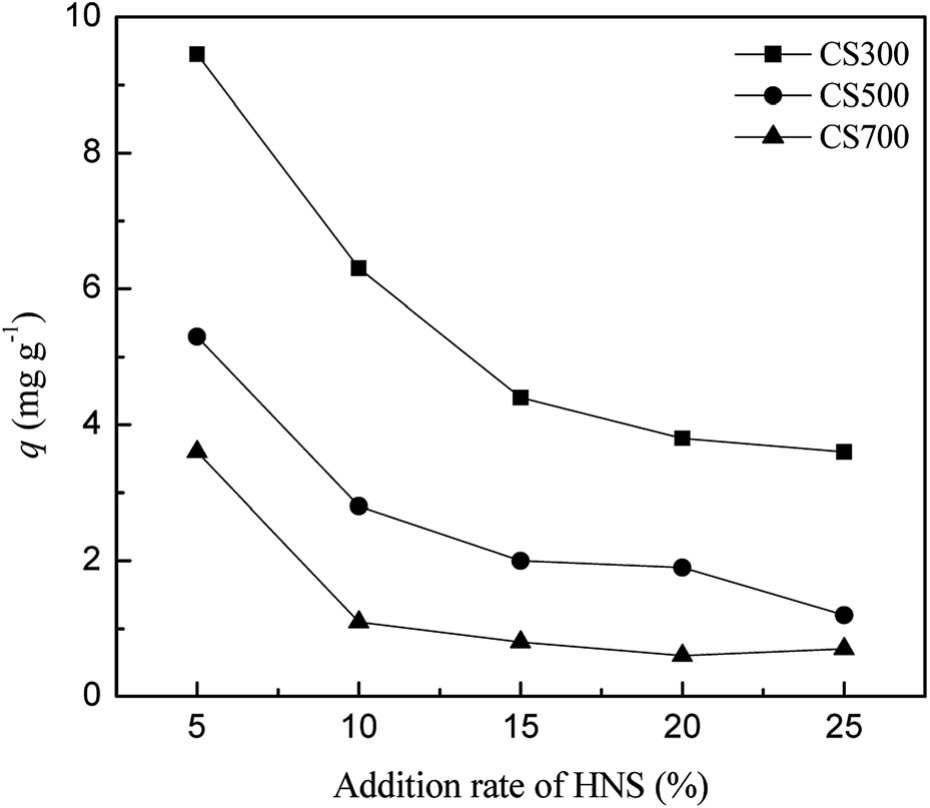
Plots of released amounts of SO_4_^2−^ in CS300, CS500 and CS700 *versus* addition rate of HNS.

If biochar is applied as one of the additives in soil to supply sulfur, it is vital to consider the effect of other nutrient elements on the release of sulfur. Thus, the influence of different additions (10%, 20%, 30%, 40% and 50%) of the modified Hoagland nutrient solution (HNS) on the release of sulfur was investigated. There was a negative correlation between the amount of released sulfate and the modified HNS concentration (Fig. 5). The drastic decreases in sulfur released from CS300 (from 9.5 to 6.3 mg g^−1^), CS500 (from 5.3 to 2.8 mg g^−1^) and CS700 (from 3.6 to 1.1 mg g^−1^) were observed when the addition rates increased from 5% to 10%. The possible reason for the decrease in sulfate release could be that the increased amount of cation (Ca^2+^) in the modified HNS reacted with SO_4_^2−^ to form CaSO_4_ on the biochar surface and are difficult to release again. Qian *et al.* found that the introduction of Hoagland nutrient solution led to the decrease in the release of P in biochars due to the formation of precipitates between dissolved P and excessive Ca^2+^ and Mg^2+^.^17^

## Conclusions

The total sulfur content in CS300 was higher than those in CS500 and CS700. The contents of inorganic sulfate also had a temperature-dependent pattern, which suggest that the pyrolysis temperature is an important factor influencing the form of sulfur in the biochar. The release kinetics could be well-fitted with a pseudo-second-order model. The introduction of Hoagland nutrient solution (HNS) led to the decrease in the release of water-soluble sulfate due to the formation of precipitates between dissolved sulfate and excessive Ca^2+^. The results indicate that low temperatures might be optimal for producing biochar from corn straw to improve the use of sulfur as a fertilizer.

## Conflicts of interest

There are no conflicts to declare.

## Supplementary Material
